# Transcriptional inhibiton of Hoxd4 expression by miRNA-10a in human breast cancer cells

**DOI:** 10.1186/1471-2199-10-12

**Published:** 2009-02-22

**Authors:** Yuliang Tan, Bo Zhang, Tao Wu, Geir Skogerbø, Xiaopeng Zhu, Xiangqian Guo, Shunmin He, Runsheng Chen

**Affiliations:** 1 National laboratory of Biomacromolecules, Institute of Biophysics, Chinese Academy of Sciences, Beijing 100101, PR China; 2 Graduate University of Chinese Academy of Sciences, Beijing, 100037, PR China

## Abstract

**Background:**

Small noncoding RNAs (ncRNAs), including short interfering RNAs (siRNAs) and microRNAs (miRNAs), can silence genes at the transcriptional, post-transcriptional or translational level [1,2].

**Results:**

Here, we show that microRNA-10a (miR-10a) targets a homologous DNA region in the promoter region of the *hoxd4 *gene and represses its expression at the transcriptional level. Mutational analysis of the miR-10a sequence revealed that the 3' end of the miRNA sequence is the most critical element for the silencing effect. MicroRNA-10a-induced transcriptional gene inhibition requires the presence of Dicer and Argonautes 1 and 3, and it is related to promoter associated noncoding RNAs. Bisulfite sequencing analysis showed that the reduced *hoxd4 *expression was accompanied by *de novo *DNA methylation at the *hoxd4 *promoter. We further demonstrated that trimethylation of histone 3 lysine 27 (H3K27me3) is involved in the miR-10a-induced *hoxd4 *transcriptional gene silence.

**Conclusion:**

In conclusion, our results demonstrate that miR-10a can regulate human gene expression in a transcriptional manner, and indicate that endogenous small noncoding RNA-induced control of transcription may be a potential system for expressional regulation in human breast cancer cells.

## Background

MicroRNAs (miRNAs) are an important small noncoding family of 19 to 26 nucleotide long endogenous RNAs that play critical roles in cognate mRNA cleavage and translational repression [1,2]. They participate in a variety of cell physiological functions such as metabolism, differentiation, morphogenesis, development and apoptosis [3]. Large numbers of miRNA have been identified in almost all genetically dissected species including animals, plants, and viruses (miRBase Release 12.0). Experimental evidence implies that miRNAs can regulate tumor susceptibility genes [4,5], and expression profiling assays have uncovered characteristic miRNA signatures in human tumors [6,7]. MicroRNA-induced transcriptional gene silencing through *de novo *DNA methylation or chromatin modification has been demonstrated in yeast and plants[8]. Although it has been reported that exogenous siRNAs can mediate transcriptional inhibition through promoter methylation in human cells and miRNA could act as a cis-regulator to modulate gene expression [9-11], transcriptional inhibition directed by endogenous small noncoding RNAs remains to be reported.

**Figure 1:**
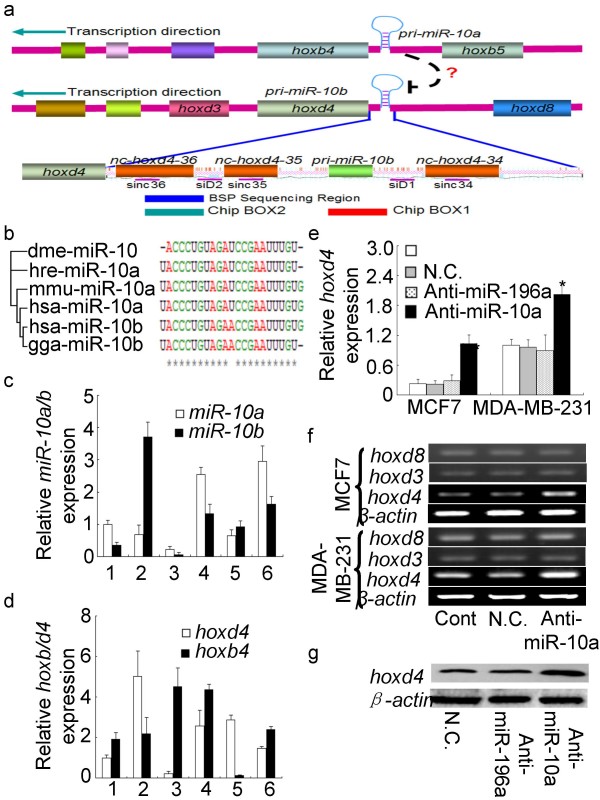
**MiR-10a-induced inhibition of *hoxd4 *gene expression**. (a) Schematic representation of the miR-10a&b loci upstream of the *hoxd4 *and *hoxb4 *genes. The insert shows the ncRNA loci, the BSP analysed region, the siRNA target sites, and the regions analysed by ChIP in the *Hoxd4 *promotor. (b) MiRNA10 exhibits high evolutionary conservation in human, mouse, zebrafish, *Drosophila *and chicken. (c) Profile of miR-10a/b expression in cancer cells by real time PCR (1: MCF7; 2: MDA-MB-231; 3: MCF10A: 4: HepG2; 5: HeLa; 6: A549). (d) Quantitative RT-PCR analysis of *hoxd4 *and *hoxb4 *gene expression in human cancer cell lines (1: MCF7; 2: MDA-MB-231; 3: MCF10A: 4: HepG2; 5: HeLa; 6: A549). The expression miR-10a varies inversely with the expression of *hoxd4 *in all six cell types. (e) Quantitative PCR analysis of the *hoxd4 *RNA levels in MCF7 and MDA-MB-231 cells treated with antisense miR-10a 2'-O-methyl oligos, miR-196a 2'-O-methyl oligos or negative control oligos (N.C.). (f) RT-PCR analysis of the *hoxd4*, *hoxd3 *and *hoxd8 *mRNA levels in MCF7 and MDA-MB-231 cells treated with antisense miR-10a 2'-O-methyl oligos or negative control oligos (N.C.). (g) Protein analysis of Hoxd4 in MDA-MB-231 cells after transfection with antisense miR-10a 2'-O-methyl oligos, miR-196a 2'-O-methyl oligos or negative control oligos (N.C.).

Homeobox genes are a group of evolutionarily conserved members that regulate animal morphological diversity at the organismal and evolutionary level [12]. Computational analyses have identified most vertebrate and invertebrate *Hox *genes as putative miRNA targets. It is believed that knowledge about the relationship between *Hox *genes and miRNAs is important for the understanding of the *Hox *gene regulatory mechanism in animal development as well as in tumor invasion and metastasis [13,14]. The human hsa-miR-10a locus maps upstream of *hoxb4 *and the hsa-miR-10b locus is similarly situated in the promoter region of *hoxd4 *(Fig. 1a). Hsa-miR-10a and hsa-miR-10b deviate in only one nucleotide located at the center of their sequence, and the miR-10 family exhibits strong evolutionary conservation across a number of animal species such as human, mouse, zebrafish, *Drosophila*/fly and chicken (Fig. 1b). The zebrafish miR-10a and miR-10b loci are not always coordinately expressed with their downstream *hox *gene suggesting that they have independent transcriptional initiation systems [14]. When expressed, the primary hsa-miR-10b transcript would be equivalent to a promoter-associated RNA [15] which could be targeted by miR-10a and thereby mediate induction of transcriptional gene silence of the *hoxd4 *locus. Here, we show that an endogenous miRNA transcriptionally modulates *hoxd4 *expression in human cancer cells through *de novo *DNA methylation.

## Results

### MicroRNA-10a inhibits *hoxd4 *gene expression

To explore whether miR-10a can suppress *hoxd4 *expression, the expression levels of the *hoxd4 *and *hoxb4 *mRNAs were compared to those of miR-10a and -10b in several human cell lines including human breast cancer cells MDA-MB-231 and MCF7, human mammary epithelial cells (MCF10A), hepatocellular liver carcinoma cells (HepG2), cervical carcinoma cells (HeLa) and lung adenocarcinoma cells (A549) (Fig. 1c, d). The *hoxd4 *expression is high in MDA-MB-231 cells, but low in MCF-7 cells and MCF10A cells (Fig. 1d). This expression pattern is similar to that of the adjacent miR-10b locus, and is consistent with the possibility that these two loci are coordinately regulated (Fig. 1c). The expression of miR-10a is higher than of miR-10b in MCF7 cells, but lower than the miR-10b expression in MDA-MB-231 cells (Fig. 1c), and the expression of miR-10a varies inversely with the expression of *hoxd4 *in all six cell types. This negative correlation indicates that miR-10a might play a role in modulating *hoxd4 *gene expression in these cells. The observation that miRNAs 10a and 10b do not always present similar expression profiles as their adjacent genes resemble similar findings in zebrafish [14] and could indicate that under some conditions the miRNAs may be regulated independently of their adjacent *hox *genes (Fig. 1c, d).

To establish that miR-10a inhibits *hoxd4 *gene expression, we first performed *in vitro *loss-of-function analyses by silencing the miRNA with antisense oligonucleotides and assessing *hoxd4 *gene expression. Quantitative PCR showed that unlike negative control 2'-O-methyl oligos (N.C.) or anti-miR-196a, the transfection of a modified 2'-O-methyl miR-10a antisense oligonucleotide (anti-miR-10a) resulted in a 2-fold increase in *hoxd4 *mRNA levels MDA-MB-231 cells and a 4-fold increase in *hoxd4 *mRNA levels in MCF7 cells(Fig. 1e). No change was observed in the expression levels of the adjacent *hoxd3 *and *hoxd8 *loci (Fig. 1f), indicating that the miR-10a-induced *hoxd4 *gene suppression did not affect other regions on the same chromosome. Nor was there any change in hoxd4 protein expression levels when MDA-MB-231 cells were transfected with negative control 2'-O-methyl oligos (N.C.) or anti-miR-196a (Fig. 1g), suggesting the observed increase in *hoxd4 *expression is a specific effect of the endogenous miR-10a.

### MicroRNA-10a decreases *hoxd4 *gene expression through transcriptional inhibition

**Figure 2:**
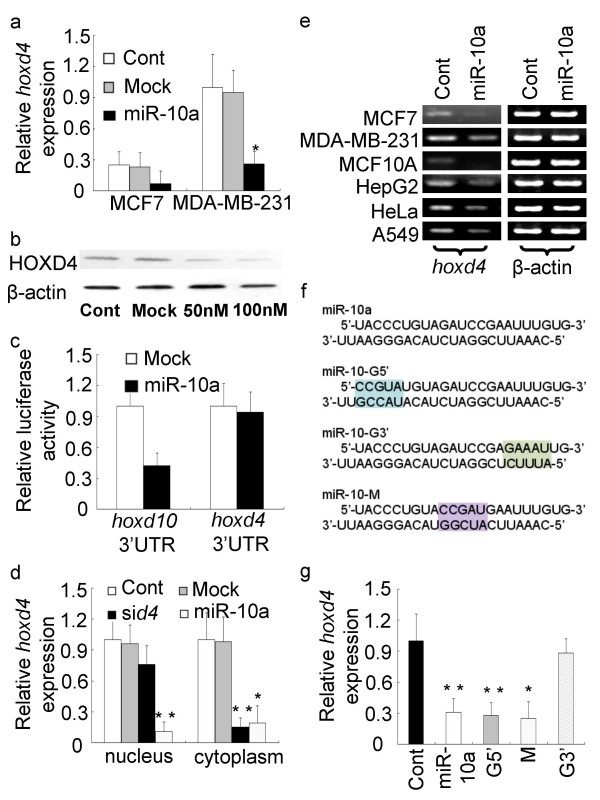
**MiR-10a modulates *hoxd4 *gene expression**. (a) Quantitative PCR analysis of *hoxd4 *gene expression in MCF7 and MDA-MB-231 cells treated with miR-10a duplexes or mock oligos. (b) Protein analysis of Hoxd4 in MDA-MB-231 cells after transfection with miR-10a. When treated with 100 nM miR-10a duplexes, the expression of Hoxd4 protein was almost abolished compared to transfection with 50 nM miR-10a duplexes or control. (c) Effect of transfection of MCF7 cells with miR-10a duplexes on the relative luciferase activity of luciferase *hoxd10 *3'UTR and *hoxd4 *3'UTR reporter constructs. (d) Nuclear run-on assay of *hoxd4 *in the presence or absence of miR-10a and *sid4*. (e) RT-PCR analysis of *hoxd4 *expression in several human cells after transfection with miR-10a. The expression of *hoxd4 *mRNA was reduced after the transfection of miR-10a duplexes in all six cell types. (f) Schematic representation of the miR-10a-G5', -M and -G3' mutants. (g) *Hoxd4 *expression in MCF7 cells transfected with miR-10a and its mutants. The 3'most portion of miR-10a sequence is most important for miR-10a induced gene silence of *hoxd4*.

To determine whether miR-10a over-expression would influence the *hoxd4 *mRNA expression level, 100 nM miR-10a duplex was transfected into MCF7 and MDA-MB-231 cells (Fig. 2a). In both cell lines, the result was a five to eight fold decrease in the *hoxd4 *mRNA levels 48 hrs after transfection. Western blot analysis of MDA-MB-231 cells showed that the Hoxd4 protein levels were consistent with the results obtained with qPCR (Fig. 2b). It has been reported that miR-10a could target the 3'UTR sequence of the *hoxd10 *gene [13], but based on current algorithms (miRanda) there are no likely miR-10a target sites in the 3'UTR of the *hoxd4 *mRNA. We assessed the levels of miRNA silencing by cloning the 3'UTRs of these two genes into a luciferase reporter. We found that transfection of 100 nM miR-10a duplexes into MCF7 cells caused a 50% decrease in luciferase activity in the reporter containing the *hoxd10 *3'UTR while no changes was detected in cells expressing the reporter with the *hoxd4 *3'UTR (Fig. 2c). However, upstream of the *hoxd4 *transcription initiation site there is a site with near perfect complementarity to the miR-10a sequence. An intriguing question is therefore whether miR-10a could regulate *hoxd4 *gene expression in a transcriptional manner. To establish that the loss of *hoxd4 *expression was due to transcriptional silencing by miR-10a, we carried out nuclear run-on experiments (Fig. 2d). *Hoxd4 *transcription was almost abolished in MCF7 nucleii when treated with miR-10a duplexes, whereas no changes were observed when MCF7 was transfected with the siRNA si*d4 *designed to target the *hoxd4 *mRNA for cleavage. When MCF7 cytoplasmic extracts were treated similarly, both miR-10a and si*d4 *significantly reduced *hoxd4 *mRNA levels, suggesting that the miR-10a induced *hoxd4 *gene downregulation is achieved at the transcriptional level.

To test whether miR-10a-induced suppression of *hoxd4 *mRNA is common in other human cell lines, MCF10A, HepG2, HeLa and A549 were also investigated. All six cell types showed a similar tendency towards reduced *hoxd4 *expression when transfected with 100 nM miR-10a (Fig. 2e). To determine which part of the miRNA sequence is most important for the observed effects, we mutated the miR-10a sequence in either the first (or 5'most) five basepairs (G5'), in the middle five basepairs (M), or in the last (or 3'most) five basepairs (G3'), and analyzed the effects of the mutant miR-10a duplexes in MCF7 cells (Fig. 2f). Transfection with miR-10a-G5' and miR-10a-M inhibited *hoxd4 *mRNA expression to the same extent as did wildtype miR-10a, however, transfection with miR-10a-G3' produced nearly no inhibition of the *hoxd4 *mRNA expression (Fig. 2g). These data indicate that miR-10a specifically decrease *hoxd4 *expression through transcriptional inhibition, and suggest that the 3'most portion of miR-10a is most important for this activity.

### MicroRNA-10a-induced transcriptional inhibition of *hoxd4 *is related to promoter-associated ncRNAs

**Figure 3:**
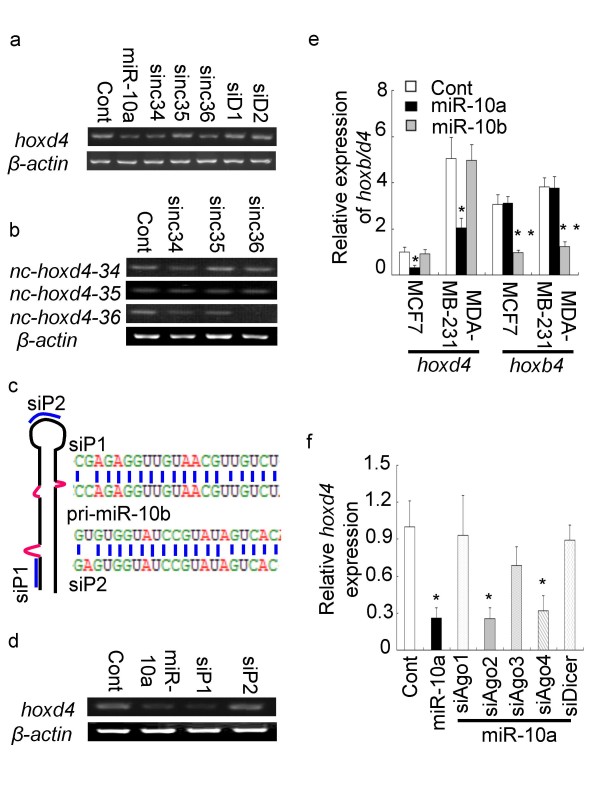
**MiR-10a-induced transcriptional inhibition of *hoxd4 *is related to promoter-associated ncRNAs**. (a) Effects of promoter target site on siRNA-induced inhibition of *hoxd4 *expression in MCF7 cells. Si-nc34 and si-nc36 inhibited *hoxd4 *mRNA expression in MCF7 cells, whereas siD1 and siD2 had almost no effect in *hoxd4 *mRNA expression. (b) RT-PCR assay of ncRNAs in the *hoxd4 *promotor region of MCF7 cells after transfection with si-nc34, si-nc35 and si-nc36. Si-nc35 did not affect the expression level of *nc-hoxd4*-*35*. (c) Schematic representation of the siP1 and siP2 target sites on hsa-miR-10b. SiP1 and SiP2 were designed to target the stem and lopp region, respectively, of has-miR-10b precusor. (d) *Hoxd4 *expression after transfection of MCF7 cells with siP1 and siP2. SiP2 couldn't affect the *hoxd4 *mRNA expression. (e) Expression of *hoxd4 *and *hoxb4 *(relative to *β-actin*) in MCF7 and MDA-MB-231 cells after transfection with miR-10a and miR-10b duplexes. MiR-10a and -10b couldn't target their own primary transcript to modulate downstream gene expression. (f) *Hoxd4 *expression in MCF7 cells after RNAi against Argonautes and Dicer mRNAs. The results suggested that Dicer, AGO1 and AGO3 are required for the induction of transcriptional inhibition by miR-10a duplexes.

The human miR-10a and miR-10b sequences deviate in only one nucleotide, and the mature miR-10a could in principle regulate *hoxd4 *by targeting the primary miR-10b transcript. Expression of intergenic ncRNA loci has been shown to influence the expression of other, nearby loci in the human genome, and several noncoding RNAs have been detected upstream of *hoxd4 *locus [15,16]. To assess whether miRNA-induced transcriptional inhibition of the *hoxd4 *locus depends on the target site being located within a promoter-associated transcript [15], several RNA duplexes complementary to the *hoxd4 *promotor were designed. The duplexes were designed to target *nc-hoxd4-34*, *nc-hoxd4*-*35*, or *nc-hoxd4*-*36 *loci [17] (si-nc34, si-nc35, si-nc36, respectively) or the non-transcribed sequence spacing these loci (siD1, siD2) (Fig. 1a). In accordance with the idea that RNA expression is required for promoter targeting, we found that two of the siRNAs targeting ncRNA loci (si-nc34 and si-nc36) in MCF7 cells inhibited *hoxd4 *expression, whereas the siRNAs targeting the intervening non-transcribed sequence siD1 and siD2 had almost no effect in *hoxd4 *expression (Fig. 3a). Further analysis of the effect of the siRNAs showed that both si-nc34 and si-nc36 clearly reduced the levels of their respective noncoding transcript targets (Fig. 3b), whereas si-nc35 did not affect the expression level of *nc-hoxd4*-*35*, possibly also explaining the failure of this siRNA to affect *hoxd4 *expression. To explore whether the secondary structure of the promoter-associated RNA target would influence the observed transcriptional inhibition, two RNA duplexes was designed to target the pri-miR-10b transcript either at the stem region (siP1) or the loop region (siP2; Fig. 3c). The siP1 duplex decreased the expression of *hoxd4 *even more strongly than did the miR-10a duplex, whereas transfection with the siP2 duplex had no affect on the *hoxd4 *expression (Fig. 3d). The data thus suggest that miRNA-induced transcriptional inhibition is mediated through interference with promoter-associated transcripts, and that the secondary structure of the promoter-associated ncRNAs can influence the inhibitory effect.

### MiR-10a/b do not silence transcription of immediate downstream genes

If the transcriptional inhibition of *hoxd4 *expression is achieved by miR-10a targeting the miR-10b primary transcript, then the possibility remains that it could also target its own primary transcript. To assess this possibility and also whether miR-10b might target the primary miR-10a transcript and thereby induce transcriptional inhibition its adjacent *hoxb4 *locus, the *hoxd4 *and *hoxb4 *mRNA levels in MCF7 and MDA-MB-231 cells were evaluated by quantitative RT-PCR. The results show that *hoxd4 *expression was reduced when transfected with the miR-10a duplexes, and similarly that *hoxb4 *was downregulated after transfection with miR-10b. Neither miR-10a nor miR-10b reduced the expression of their respective immediate downstream *hox *locus, possibly indicating that none of these miRNAs are able to target their own primary transcripts (Fig. 3e). MiR-10a/b and miR-320 are both encoded in the promoter region of genes, and the observation that miR-320 has as a cis-regulatory role may be very important in miRNA research[11]. Although, there are no negative correlations between the expression of the miRNAs and their respective host genes, and miRNAs target their homologous pre-miRNA sites to modulate gene expression would be also another interesting research topic in molecular biology.

### Dicer and AGO1/3 proteins are involved in miR-10a induced transcriptional gene inhibition

Previous studies have shown that siRNAs targeting selected promoter regions of human genes require the recruitment of the Argonaute 1 (Ago1) and Argonaute 2 (Ago2) [18,19]. Also knockdownofDicer relieve the transcriptional inhibition and attenuate abnormal promoter methylation in human cells [20]. We decided to test whether transcriptional inhibition induced by endogenous small noncoding RNAs is Ago-dependent, and also whether Dicer is involved in this process. Specific siRNAs targeting Agos 1–4 (siAgo1, siAgo2, siAgo3 and siAgo4) and Dicer (siDicer) were shown to knock-down the corresponding Argonaute and Dicer mRNA efficiently [19,21]. Each of these siRNAs was transfected into MCF7 cells, either alone or in combination with the miR-10a duplex. Co-transfected with the miR-10a duplex, siAgo1 or siDicer almost completely prevented the reduction in *hoxd4 *expression induced by miR-10a (Fig. 3f). SiAgo3 produced a similar, but less pronounced effect. These data show that AGO1 and AGO3 are required for the induction of transcriptional inhibition by endogenous small noncoding RNAs. The effect of Dicer knockdown further suggests involvement of this protein in DNA methylation of the *hoxd4 *promotor.

### MicroRNA-10a-induced inhibition of transcription is associated with DNA methylation of the *hoxd4 *promotor

**Figure 4:**
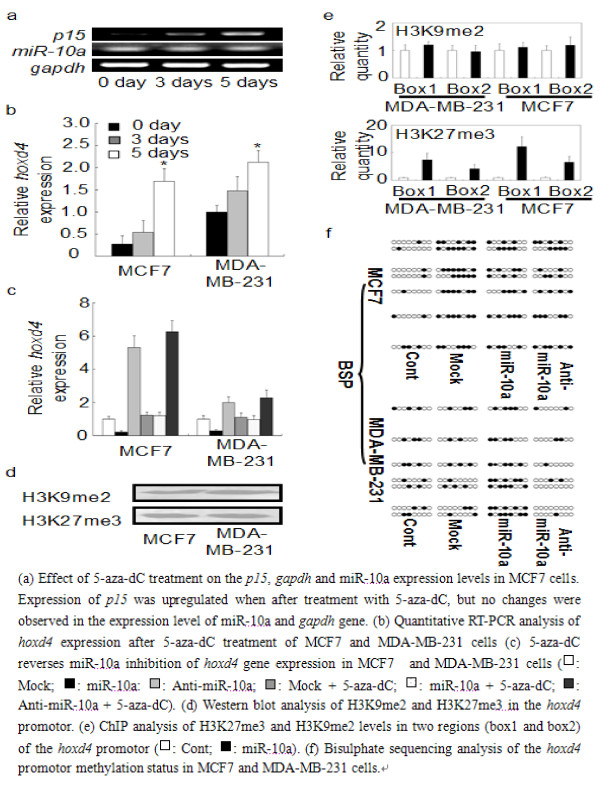
**MiR-10a-induced transcriptional gene inhibition is associated with DNA methylation of the *hoxd4 *promoter**. (a) Effect of 5-aza-dC treatment on the *p15*, *gapdh *and miR-10a expression levels in MCF7 cells. Expression of *p15 *was upregulated when after treatment with 5-aza-dC, but no changes were observed in the expression level of miR-10a and *gapdh *gene. (b) Quantitative RT-PCR analysis of *hoxd4 *expression after 5-aza-dC treatment of MCF7 and MDA-MB-231 cells (c) 5-aza-dC reverses miR-10a inhibition of *hoxd4 *gene expression in MCF7 and MDA-MB-231 cells (□: Mock; ■: miR-10a: : Anti-miR-10a; : Mock + 5-aza-dC; □: miR-10a + 5-aza-dC; : Anti-miR-10a + 5-aza-dC). (d) Western blot analysis of H3K9me2 and H3K27me3 in the *hoxd4 *promotor. (e) ChIP analysis of H3K27me3 and H3K9me2 levels in two regions (box1 and box2) of the *hoxd4 *promotor (□: Cont; ■: miR-10a). (f) Bisulphate sequencing analysis of the *hoxd4 *promotor methylation status in MCF7 and MDA-MB-231 cells.

Transcriptional silencing is commonly associated with epigenetic chromatin modifications. To test whether miR-10a suppresses *hoxd4 *expression by *de novo *DNA methylation, MCF7 and MDA-MB-231 cells were treated for 5 days with 0.75 μM 5-Aza-2'-deoxycytidine (5-aza-dC), a DNA methylotransferase (DNMT) inhibitor. In MCF7 cells, the 5-aza-dC treatment clearly upregulated the expression of the *p15 *gene, whose promotor is normally methylated, but did neither affect the expression of the unmethylated *gapdh *gene nor affect the expression of miR-10a (Fig. 4a). Treatment with 5-aza-dC increased the *hoxd4 *mRNA level about 5-fold in MCF7 cells, compared to only 2-fold in MDA-MB-231 cell lines for 5 days (Fig. 4b). To show that miR-10a can silence *hoxd4 *expression by promoter methylation, MCF7 cells and MDA-MB-231 cells were transfected with either the miR-10a duplexes or anti-miR-10a, and then treated with 1 μM 5-aza-dC for 48 h. We found that after inhibiton of the DNA methylotransferase activity, miR-10a could no longer silence *hoxd4 *mRNA expression (Fig. 4c).

Targeting promoters with siRNAs have been shown to relative with the facultative heterochromatin marks H3K9me2 and H3K27me3 in human cells [9,18]. To determine whether miR-10a could induce these repressive histone modifications, we used chromatin immunoprecipitation (ChIP) to screen the *hoxd4 *promoter for H3K9me2 and H3K27me3 at regions overlapping the miR-10a target site (Chip Box1: -1081 ~ -972) and the bisulphite sequencing PCR (BSP) detection site (Chip Box2: -330 ~ -122). Western blot analysis demonstrated the presence of H3K9me2 and H3K27me3 in the promoter region of the *hoxd4 *gene (Fig. 4d). After transfection with miR-10a, an increase in H3K27me3 in the two analysed regions (Box1 and Box2) was observed in both MCF7 cells and MDA-MB-231 cells (Fig. 4e). We next analysed the DNA methylation status by performing bisulphite sequencing of the *hoxd4 *promoter. Aberrant methylation is often observed in cancer cells. CpG and CpNpG methylation are both strongly correlated with gene silencing in plants. CpNpG methylation is rare in the human genome, and no functional implications for gene silencing have ever been documented[22]. Consistents with previous studies [22,23], we found no CpNpG methylation in the our bisulfite-sequenced region (BSP Sequencing Region: -330 ~ -122). Cultures treated with mock duplexes presented CpG methylation in this region, but consistent with the observation that the *hoxd4 *mRNA level is lower in MCF7 than in MDA-MB-231 cell lines, the *hoxd4 *promoter region was clearly more densely methylated in MCF7 cells than in MDA-MB-231 cells. When the cultures were transfected with miR-10a duplexes, the extent of methylation increased compared to cultures treated with control RNA duplexes. Contrarily, when cells were treated with 2'-O-methyl-miR-10a (anti-miR-10a), we found that the extent of methylation decreased and was accompanied by upregulation *hoxd4 *expression in both cells types (Fig. 4f). These results thus indicate that the miR-10a-induced inhibition of *hoxd4 *transcription is accompanied by *de novo *DNA methylation and H3K27me3 formation in the targeted promoter region.

## Discussion

We demonstrate that an endogenous small noncding RNA involved in transcriptional gene regulation in human cells. This observation gives new insights into the mechanisms by which an increase in the miR-10a expression leads to a concomitant reduction in the *hoxd4 *expression. Bidirectional transcription leading to two functionally different miRNAs originating from the same genomic locus has been reported in flies [24-26]. Here, we show that two miRNAs, miR-10a and miR-10b, mapping to two different chromosomes, can target each other's primary transcipts to repress the expression of neighboring *hox *genes. Antisense RNA-mediated gene silencing and *de novo *methylation of CpG islands in the promoter have been demonstrated in humans [27]. However, the molecular mechanisms by which small RNAs mediate DNA methylation of targeted promoter regions in the human genome remains to be elucidated. Some groups have observed siRNA-directed DNA methylation at targeted promoters [9,10,28,29] whereas others have not found such an effect [19,22]. A recent report has showed that miR-10a could bind the 5'UTR of ribosomal protein mRNAs and enhance their translation [30]. One possible explanation for this observation might that non-coding RNAs are expressed in the target region. It has been demonstrated that promoter-associated noncoding RNAs can be recognized by miRNAs and direct epigenetic silencing complexes to the corresponding targeted promoters, thereby mediating transcriptional inhibition [15]. In the case of the *hoxd4 *locus, the adjacent pri-miR-10b could serve as a promoter-associated non-coding RNA, mediating induction of transcriptional silencing when targeted by miR-10a.

Recently, Dicer has been reported to attenate abnormal promoter DNA methylation in cancer cells [20]. Our results define the first case in which an endogenous miRNA targets and methylates a promoter region through Dicer action. In plants, methylation is a crucial step in microRNA biogenesis, and depends on HEN1, a methyltransferase that adds a methyl group to the 3'-most nucleotide of small non-coding RNAs in both plants and mammals [31,32]. miRNAs that regulate *Rbl2*-dependent DNMT expression in mouse embryonic stem cells have also been detected [29,33]. Ago1 and Ago4 has been shown to be involved in siRNA-directed chromatin modification, including histone methylation and non-CpG DNA methylation in plants and yeast [34,35]. Ago1 is required for both siRNA-mediated transcriptional gene silencing and the recruitment of histone methyltransferase activity to H3K9me2 and H3K27me3 at a siRNA-targeted promoters in human cells [18], and Ago3 has the ability to interact with methyltransferases [36]. Here we show that Ago 1 and Ago3 participate in miRNA-mediated *de novo *DNA methylation in human cancer cells, further implicating both the miRNA machinery and the chromatin remodelling complexes in RNA directed transcriptional gene silencing.

## Conclusion

Taken together, our data support the notion that an RNA-operated system is involved in transcriptional regulation of genes in human cells. Endogenous small non-coding RNAs might control or fine-tune gene expression at both the transcriptional and post-transcriptional levels. An understanding of such a regulatory system could prove valuable in targeted approaches to specific control of gene expression and treatment of human cancers.

## Methods

### Cell culture and transfection

Human breast cancer cell lines MCF-7, MDA-MB-231, human mammary epithelial cell line MCF10A, human cervical carcinoma cell line HeLa, human hepatocellular liver carcinoma cell line HepG2, human lung adenocarcinoma cell line A549 were obtained from the American Type Culture Collection (Rockville, MD). MCF10A cells were cultured in DMEM-F12 (Life Technologies) supplemented with 5% horse serum, 0.5 μg/ml hydrocortisone (Sigma), 10 μg/ml insulin (Sigma), 20 ng/ml epidermal growth factor (Sigma), 100 μg/ml penicillin and 100 μg/ml streptomycin. Other cells were grown in DMEM (Life Technologies) supplemented with 100 μg/ml penicillin, 100 μg/ml streptomycin and 10% heat-inactivated FBS at 37°C in a humidified atmosphere containing 5% CO_2_. Introduction of plasmids into tumor cells (3.5 × 10^6^) was performed with lipofectamine 2000 (Invitrogen) according to the manufacturer's instructions.

### RNA isolation and miRNA detection

Total RNA from cultured cells was isolated using the mirVana miRNA Isolation Kit (Ambion). Detection of the mature form of miRNAs was performed using the Hairpin-it miRNAs qPCR Quantitation Assay, according to the manufacturer's instructions (GenePharma). The *U6 *small nuclear RNA was used as an internal control.

### Western blotting

Total protein (40 μg) was resolved with 10% SDS-polyacrylamide gel eletrophoresis and bands of protein transferred to a polyvinylidene difluoride (PVDF) membrane (Amersham). The membrane was blocked with 5% nonfat milk TBS buffer overnight at RT, and incubated for 2 hours with primary antibodies. β-actin was used as loading control. The antibodies used included hoxd4 (Abcam), β-actin (SantaCruz Biotechnology). The membranes then were incubated for 1 h with HRP-conjugated goat anti-mouse (Zymed Laboratories) or rabbit anti-goat (SantaCruz Biotechnology) secondary antibody. Immunocomplexes were visualized with an ECL kit (Pierce).

### Methylation analysis and sodium bisulphite DNA sequencing

Genomic DNA (1 μg) was treated with sodium bisulphate and. used to PCR-amplify the *hoxd4 *promoter regions (-300 bp – 122 bp). The amplifed product was purified using a Qiagen PCR purification kit (Qiagen) and sequenced using the sense primer with an ABI automated fluorescent sequencer according to the manufacturer's instructions.

### Treatment of cells with 5'-aza-2'-deoxycytidine (5-aza-dC)

MCF7 and MDA-MB231 breast cancer cells were treated with 0.75 μM 5-aza-dC (Sigma), and collected 0, 3 and 5 days later. MCF7 and MDA-MB231 breast cancer cells that had been transfected with 100 nM small RNA duplexes were treated with 1 μM 5-aza-dC for 48 h after transfection before subjected to qPCR analysis.

### qRT-PCR

We extracted total RNA from the treated cells with Trizol (Invitrogen) and treated it with DNase (Qiagen). We carried out qRT-PCR analysis using QuantiTeck SYBR Green PCR kit (Qiagen). PCR primers used in this paper are listed in Table 1, Additional file 1. Real-time PCR was performed with the LightCycler 2.0 (Roche).

### Nuclear run-on assay

Nuclear run-on assays were performed in accordance with the Current Protocols of Molecular Biology. Digoxinum linked dUTP was added to an *in vitro *transcription reaction and precipitated with digoxinum antibody. Amplified β-*actin *served as a loading control. Bound RNA was eluted from the beads by adding Trizol (Invitrogen) to the beads, followed by RNA extraction and RT-real time PCR as described previously.

### Chromatin immunoprecipitation

We cross-linked DNA and processed it in accordance with the UpState Chromatin Immunoprecipitation (ChIP) Assay Kit protocol (UpState 17-295). We used 2 × 10^6 ^cells for each immunoprecipitation reaction, and used rabbit antibody to H3K27me3 (Upstate 07-449) for specific immunoprecipitation of the histone residues. The precipitates were analyzed by real-time PCR with two sets of *hoxd4 *promoter specific primers spanning the CpG island of interest (Table 1, Additional file 1).

### Luciferase reporter assay

Cells of 50% confluence in 24-well plates were transfected using Lipofectamine 2000 (Invitrogene). Firefly luciferase reporter gene constructs (200 ng) and pRL-SV40 Renilla luciferase construct (1 ng; for normalization) were cotransfected per well. Cell extracts were prepared 48 h after transfection, and the luciferase activity was measured using the Dual-Luciferase Reporter Assay System (Promega).

### Statistical Analysis

All experiments were repeated 3 times. Data are presented as mean ± s.e.m. Student's t test (two-tailed) was used to compare two groups (P < 0.05 was considered significant). Two asterisks indicated that the p-value was less than 0.01, one asterisks indicated that the p-value was less than 0.05.

## Authors' contributions

YT carried out experiments, data analysis, and drafted the manuscript. BZ developed experimental methods. TW, GS, XZ, XG and HS participated in conception and design of the study. RC supervised the study, contributed to the data analysis, and reviewed the manuscript. All authors read and approved the final manuscript.

## Supplementary Material

Additional File 1 **Tables.** Tables 1 and 2. Click here for file
