# Supplementary material for: Transcriptional inhibiton of Hoxd4 expression by miRNA-10a in human breast cancer cells
Source: BMC Mol Biol. 2009 Feb 22;10:12. doi: 10.1186/1471-2199-10-12 (PMC2680403; doi:10.1186/1471-2199-10-12)
Supplement: Additional File 1 — Tables. Tables 1 and 2. [file 1471-2199-10-12-S1.doc]

**Supplementary Material**

**Supplementary table 1. Primers and siRNAs**

| Gene | Sequence(5’—3’) | Method |
| --- | --- | --- |
| *GAPDH* | ACCACAGTCCATGCCATCAC | RT-PCR |
|  | TCCACCACCCTGTTGCTGTA | RT-PCR |
| *Hoxd4* | TCACACCCTGTGTCTGTCG | Real-time PCR |
|  | CCCCACTTCTATAAGGTCGTCAG | Real-time PCR |
| *Hoxb4* | CTCGACACCCGCTAACAAATG | Real-time PCR |
|  | TTCTGGCCCTCAGTGAATGG | Real-time PCR |
| *Hoxd3* | AGGATCCTGGTCTGAAACTCAGAGC | RT-PCR |
|  | ACTCGAGTTCATCCGCCGGTTCTGGAACCA | RT-PCR |
| *Hoxd8* | GATAACTTACAGAGACAGCCGATTTTTAC | RT-PCR |
|  | CCAATATTACCACTGGACGATTTACA | RT-PCR |
| *P15* | CCTTAAATGGCTCCACCTGC | RT-PCR |
|  | CGTTGGCAGCCTTCATCG | RT-PCR |
| *Muc-1* | AGACGTCAGCGTGAGTGATG | Real-time PCR |
|  | CAGCTGCCCGTAGTTCTTTC | Real-time PCR |
| *β-Actin* | CTCTTCCAGCCTTCCTTCCTG | Real-time PCR |
|  | CTGCTTGCTGATCCACATCTG | Real-time PCR |
| *Nc-hoxd4-34* | GGATATTCCCCTCTCGCTTC | RT-PCR |
|  | CAAGGCTGGCGGCTTTATTA | RT-PCR |
| *Nc-hoxd4-35* | AAATGGGGGCTTGCTTGTAT | RT-PCR |
|  | GCACCTCCACCTTGTCAAAA | RT-PCR |
| *Nc-hoxd4-36* | CCCACCTTTCATTGCTCTGT | RT-PCR |
|  | CCACTGATTTTGCCAACCTT | RT-PCR |
| *Dicer* | ATGAAAAGCCCTGCTTTGCAACCCCT | Real-time PCR |
|  | AGTTGCAGTTTCAGCATTACTCTT | Real-time PCR |
| *Ago1* | GCGAATTGGGAAGAGTGGTA | Real-time PCR |
|  | GCAGGTGCTGGGATAGAGAC | Real-time PCR |
| *Ago2* | CGCGTCCGAAGGCTGCTCTA | Real-time PCR |
|  | TGGCTGTGCCTTGTAAAACGCT | Real-time PCR |
| *Ago3* | ATCCCAGCTGGAACAACAGT | Real-time PCR |
|  | GCGTACGTAAGTGTGGCAGA | Real-time PCR |
| *Ago4* | GGGTAGGGAAAAGTGGCAAT | Real-time PCR |
|  | GAGCGAGTGCACCTCACATA | Real-time PCR |
| *Hoxd4* | TGGGTGATTTTGATGAAGATAAAG | BSP |
|  | CACCAAATAAACACAACTCAAAAAA | BSP |
| *Box1* | CCAGAGGTTGTAACGTTGTCTATA | CHIP |
|  | TGAAGTTTTTGCATCGACCATATA | CHIP |
| *Box2* | TGGGTGATTTTGATGAAGATAAAG | CHIP |
|  | CACCAAGTAGGCACAGCTCAAGGG | CHIP |
| *Hsa-miR-10a* | 5’-GGCTACCCTGTAGATCC-3’ | Real-time PCR |
|  | 5’-CATCAGATGCGTTGCGTA-3’ | Real-time PCR |
|  | FAM-5’CGCAGGCGCCACAAATTCCCTGCG-3’-DABCYL | Probe |
| *Hsa-mir-10b* | 5’-GGCTACCCTGTAGAACC-3’ | Real-time PCR |
|  | 5’-CATCAGATGCGTTGCGTA-3’ | Real-time PCR |
|  | FAM-5’CGCAGGCGCCACAAATTCCCTGCG-3’-DABCYL | Probe |
| siAgo1 | GAGAAGAGGUGCUCAAGAAUU | Sense strand |
|  | UUCUUGAGCACCUCUUCUCUU | Antisense strand |
| siAgo2 | GCACGGAAGUCCAUCUGAAUU | Sense strand |
|  | UUCAGAUGGACUUCCGUGCUU | Antisense strand |
| siAgo3 | GAAAUUAGCAGAUUGGUAAUU | Sense strand |
|  | UUACCAAUCUGCUAAUUUCUU | Antisense strand |
| siAgo4 | GGCCAGAACUAAUAGCAAUUU | Sense strand |
|  | AUUGCUAUUAGUUCUGGCCUU | Antisense strand |
| Mock | UUCUCCGAACGUGUCACGUUU | Sense strand |
|  | ACGUGACACGUUCGGAGAAUU | Antisense strand |
| sinc34 | 5'-UUUCGCUUGGUCGGGCAGUAGGA-3' | Sense strand |
|  | 3'-ACCGAACCAGCCCGUCAUCCUUU-5’ | Antisense strand |
| sinc35 | 5'-UUACCUUCAAAGAAGCCCAGUGG-5' | Sense strand |
|  | 3'-UCGAAGUUUCUUCGGGUCACCUU-5' | Antisense strand |
| sinc36 | 5'-UUUCAGCUGUGCCUACUUGGUGU-3' | Sense strand |
|  | 3'-ACUCGACACGGAUGAACCACAUU-5' | Antisense strand |
| siD1 | 5'-UUAGGGUGGUGGCUCAGAGGAAG-3' | Sense strand |
|  | 3'-UGCCACCACCGAGUCUCCUUCUU-5' | Antisense strand |
| siD2 | 5’-UUGCCUAAUUUGAUUCAAGCUGG-3’ | Sense strand |
|  | 3’-CCGAUUAAACUAAGUUCGACCUU-5’ | Antisense strand |
| siP1 | 5'-UUCGAGAGGUUGUAACGUUGUCU-3' | Sense strand |
|  | 3'-GGUCUCCAACAUUGCAACAGAUU-5' | Antisense strand |
| siP2 | 5'-UUGAGUGGUAUCCGUAUAGUCAC-3' | Sense strand |
|  | 3'-CACACCAUAGGCAUAUCAGUGUU-5' | Antisense strand |
| siDicer | 5'-UGCUUGAAGCAGCUCUGGAUU-3' | Sense strand |
|  | 5'-UCCAGAGCUGCUUCAAGCAUU-3' | Antisense strand |
| N.C. | 5’-CAG UAC UUU UGU GUA GUA CAA-3’ | 2’-O-methyl |
